# Health Care Costs and Treatment Patterns Associated with Uterine Fibroids and Heavy Menstrual Bleeding: A Claims Analysis

**DOI:** 10.1089/jwh.2020.8983

**Published:** 2022-06-14

**Authors:** Alice Wang, Siting Wang, Charlotte D. Owens, Jamie B. Vora, Michael P. Diamond

**Affiliations:** ^1^ AbbVie, Inc., North Chicago, Illinois, USA.; ^2^ Department of Obstetrics and Gynecology, Augusta University, Augusta, Georgia, USA.

**Keywords:** uterine fibroids, heavy menstrual bleeding, costs

## Abstract

**Background:**

Heavy menstrual bleeding (HMB) is one of the most common distressing complications of uterine fibroids (UF); however, data on the health care costs for treatments in women experiencing HMB associated with UF are lacking. The objective of this study was to compare the direct costs and treatments patterns for women diagnosed with UF+HMB, UF only, and HMB only in the United States.

**Materials and Methods:**

The study design was retrospective matched cohort study using claims data. Women, aged 18–51 years, comprising four cohorts (HMB only, UF only, UF+HMB, and controls) were identified in the IBM MarketScan^®^ Commercial Claims and Encounters Database (October 1, 2007‒September 30, 2018) and matched by demographics and Charlson Comorbidity Index score. Baseline characteristics and treatments during the 12 months post-diagnosis were summarized descriptively. Costs (2018 U.S. dollars) during the post-diagnosis year were compared using analysis of variance.

**Results:**

Before matching, women with UF+HMB represented 54% of UF cases. Following diagnosis, 32% in the matched UF+HMB cohort had no treatment, 49% underwent surgeries/procedures with (12%) or without (37%) medications, and 18% received medications only. The mean all-cause total costs for UF+HMB ($16,762) exceeded that for UF only by 24% ($13,506) and HMB only by 50% ($11,135), and almost tripled the mean cost for the control cohort ($6,691) (all, *p* < 0.001). The mean diagnosis-related costs were significantly higher for UF+HMB ($8,741) than for UF only ($4,550) and HMB only ($3,081) (all, *p* < 0.0001). Surgery/procedure costs comprised 80% of diagnosis-related medical costs for UF+HMB.

**Conclusions:**

UF with HMB were associated with significant economic burden, driven primarily by surgical/procedural costs and treatment patterns.

## Introduction

Uterine fibroids (UF), also known as leiomyomas, represent the most common benign gynecological tumors.^1^ In the United States, the cumulative incidence of UF by age 50 approaches 70% among white women and exceeds 80% among black women.^2^ While up to 75% of cases are asymptomatic, heavy menstrual bleeding (HMB) occurs in about one third of patients and can lead to life-threatening anemia^3^ and impair quality of life.^4^ The economic consequences of UF are substantial. A 2012 review of published cost estimates cited annual direct and indirect costs ranging from $5.9 to $34.4 billion for women seeking treatment for UF in the United States.^5^ UF is the primary cause of hospitalization related to a gynecological disorder^6^ and accounts for up to 50% of hysterectomies.^7–9^ A retrospective cohort study in the United States also implicated UF as the cause of bleeding in nearly half (48%) of hospitalized women discharged with HMB and extremely low hemoglobin levels.^10^

Numerous studies have quantified the direct costs of UF in the United States using different methods and cost measures.^11–13^ Most studies have focused on the costs for surgery or inpatient care for UF, primary cost drivers in this population; ^12,13^ however, none have examined costs in the context of treatment practices or the absence or presence of HMB. To address this gap in real-world evidence, this study employed a large administrative claims database to examine the health care costs and treatment patterns for mutually exclusive, matched cohorts of women diagnosed with UF only, HMB only, or both UF and HMB (“UF+HMB”), and neither diagnosis. The primary aim was to estimate the direct health care costs and treatment patterns associated with these distinct clinical conditions while assessing the respective cost burden to the U.S. health care system.

## Materials and Methods

### Study design and population

This retrospective cohort study analyzed data from the IBM MarketScan^®^ Commercial Claims and Encounters Database (IBM Watson Health, Cambridge, MA) for the period October 1, 2007, through September 30, 2018. MarketScan provides electronic claims data submitted by large employers, managed care organizations, and hospitals in the United States for more than 160 million covered lives. Cases and UF/HMB diagnosis-specific claims were identified by *International Classification of Diseases, Ninth (“ICD-9”) and Tenth (“ICD-10”) Revision, Clinical Modification codes*. Prescription drug claims were identified by National Drug Codes and Healthcare Common Procedure Coding System codes. All database records are statistically de-identified and certified to be fully compliant with U.S. patient confidentiality requirements set forth in the Health Insurance Portability and Accountability Act of 1996. Because this study used only de-identified patient records and did not involve the collection, use, or transmittal of individually identifiable data, institutional review board approval to conduct this study was not necessary.

Four cohorts were considered eligible based on diagnosis: (1) UF only, (2) HMB only, (3) UF+HMB, and (4) a control cohort with neither diagnosis. Eligible women in the UF only cohort had ≥1 diagnosis of UF (ICD-9 218.x or ICD-10 D25.x) but no diagnosis of HMB (ICD-9 626.2 or 627.0, or ICD-10 N92.0, N92.1, or N92.4); those in the HMB only cohort had ≥1 diagnosis for HMB, but none for UF. Women in the UF+HMB cohort had diagnoses for both UF and HMB, whereas those eligible for the control cohort had neither diagnosis during the study period. The index date represented the date of first UF diagnosis for women with UF only or the date of the first HMB diagnosis for women with HMB only. For the UF+HMB cohort, the index date was the later date of first diagnosis of UF or HMB. Women in the control cohort were assigned random index dates from a uniform distribution within the same period.

Eligible women were included if they were aged 18–51 years on the index date and continuously enrolled for ≥1 year before the index date (“pre-index” baseline period) and ≥1 year following the index date (“post-index” follow-up). Women who had a diagnosis of gynecological cancer during the study period or a pre-index history of hysterectomy, myomectomy, uterine artery embolization, endometrial ablation, or magnetic resonance-guided high-intensity focused ultrasound were excluded.

### Study outcomes

Outcomes were analyzed in the four cohorts of women matched 1:1:1:1 by age, geographical region of residence, insurance plan, and Charlson Comorbidity Index (CCI) scores.^14^ The primary outcome was direct all-cause and diagnosis-related health care costs incurred during the 1-year post-index period. Total costs were estimated as the sum of medical (inpatient, outpatient, emergency room, and other costs) plus pharmacy costs. “Other costs” pertained to costs for health services rendered in a residential/patient home, rural health clinic, urgent care facility, independent clinic, or unlisted facility. All costs represented gross payments to providers adjusted to 2018 U.S. dollars using the Consumer Price Index. Diagnosis-related costs only included those which had at least one UF or HMB diagnostic codes associated with the same observation.

Treatments analyzed focused on either surgeries and procedures (hysterectomy, endometrial ablation, myomectomy, uterine artery embolization, and magnetic resonance-guided high-intensity focused ultrasound) or medications. Medication use included contraceptives, gonadotropin-releasing hormone agents, non-steroidal anti-inflammatory drugs, or antifibrinolytic agents.

Patient demographics as of the index date and the CCI and the frequency of anemia diagnoses during the 1-year pre-index period before matching were reported. Outcomes analyzed in the matched cohorts included the pre- and post-index frequencies of anemia diagnoses, contraceptive use, inpatient admissions, and treatments (no treatment, medications only, surgeries/procedures only, and medications with surgeries/procedures).

### Statistical analyses

Statistical analyses were conducted using SAS, Version 9.4 (Cary, NC). Patient demographics, clinical attributes, treatment utilization and patterns, and costs are summarized descriptively as the mean ± standard deviation for continuous variables or frequency (*n* and proportion) for categorical variables. Analysis of variance (ANOVA) was used to compare the 1-year post-index costs among the four matched cohorts. A *p*-value <0.05 indicated statistical significance.

## Results

### Patient characteristics

Before matching, the study sample consisted of 3,377,957 women (mean age, 36.7 years), including 237,070 (7%) with UF only, 623,810 (18%) with HMB only, 280,636 (8%) with UF+HMB, and 2,236,441 (66%) with neither diagnosis (Supplementary Appendix Fig. SAF1 and Table 1). Women diagnosed with UF+HMB represented over half (54%) of all women with UF (total *n* = 517,706) and nearly one third (31%) of those with HMB (total *n* = 904,446). Women with UF, regardless of a diagnosis of HMB, were on average older. More than 70% were aged ≥39–51 years in the UF+HMB and UF only cohorts compared with less than 50% in the HMB only and control cohorts.

**Table 1.** Baseline Characteristics of the Study Cohorts Pre- and Post-Matching

Characteristic^a^	Pre-matching				Post-matching
	Controls	HMB only	UF only	UF+HMB	
Patients, total *n*	2,236,441	623,810	237,070	280,636	209,248
Age, years, mean ± SD	35.4 ± 10.3	36.6 ± 9.2	41.9 ± 6.9	42.9 ± 5.7	42.6 ± 6.2
Age group, years, *n* (%)					
18‒24	489,751 (21.90)	95,223 (15.26)	3,159 (1.33)	1,525 (0.54)	1,395 (0.67)
25‒31	343,803 (15.37)	79,580 (12.76)	17,707 (7.47)	10,016 (3.57)	9,673 (4.62)
32‒38	436,607 (19.52)	149,155 (23.91)	48,997 (20.67)	47,664 (16.98)	41,242 (19.71)
39‒45	470,147 (21.02)	181,716 (29.13)	77,928 (32.87)	115,601 (41.19)	75,417 (36.04)
46‒51	496,133 (22.18)	118,136 (18.94)	89,279 (37.66)	105,830 (37.71)	81,521 (38.96)
Geographical region, *n* (%)					
Midwest	510,600 (23.1)	143,654 (23.4)	41,745 (17.9)	53,034 (19.1)	38,141 (18.2)
Northeast	334,705 (15.2)	87,192 (14.2)	48,654 (20.8)	43,461 (15.7)	39,163 (18.7)
South	918,559 (41.6)	268,449 (43.7)	100,705 (43.1)	130,834 (47.2)	93,643 (44.8)
West	442,929 (20.1)	115,217 (18.8)	42,801 (18.3)	49,703 (17.9)	38,301 (18.3)
Payer type, *n* (%)					
CDHP	202,887 (9.1)	55,934 (9.0)	18,963 (8.0)	25,932 (9.2)	17,345 (8.3)
Comprehensive	35,206 (1.6)	9,339 (1.5)	3,162 (1.3)	4,096 (1.5)	2,723 (1.3)
EPO	27,382 (1.2)	7,731 (1.2)	4,198 (1.8)	3,758 (1.3)	2,938 (1.4)
HDHP	121,483 (5.4)	31,828 (5.1)	10,390 (4.4)	11,939 (4.3)	9,014 (4.3)
HMO	338,861 (15.2)	89,428 (14.3)	38,225 (16.1)	44,972 (16.0)	33,834 (16.2)
POS	171,605 (7.7)	45,927 (7.4)	19,561 (8.3)	23,413 (8.3)	17,335 (8.3)
POS with capitation	11,651 (0.5)	3,333 (0.5)	1,729 (0.7)	1,849 (0.7)	1,238 (0.6)
PPO	1,293,179 (57.8)	370,504 (59.4)	137,198 (57.9)	160,470 (57.2)	121,782 (58.2)
Unknown	34,187 (1.5)	9,786 (1.6)	3,644 (1.5)	4,207 (1.5)	3,039 (1.5)
CCI, mean ± SD	0.1 ± 0.5	0.2 ± 0.6	0.3 ± 0.8	0.2 ± 0.7	0.2 ± 0.6
Anemia diagnosis, *n* (%)	77,107 (3.4)	55,082 (8.8)	19,855 (8.4)	58,562 (20.9)	

^a^ Age, geographical region, payer type, anemia diagnosis, and contraceptive use were assessed as of the index date. CCI was measured over the 1-year pre-index period.

CCI, Charlson Comorbidity Index; CDHP, consumer directed health plan; EPO, exclusive provider organization; HDHP, high-deductible health plan; HMB, heavy menstrual bleeding; HMO, health maintenance organization; POS, point of service; PPO, preferred provider organization; SD, standard deviation; UF, uterine fibroids.

A total of 836,992 women (mean age, 42.6 ± 6.2 years)—209,248 from each cohort—were matched and included in the analyses. With the exception of the control cohort, the frequencies of anemia among the cohorts were higher during the post-index period. The UF+HMB cohort featured the highest proportions diagnosed with anemia during both the pre-index and post-index periods (20% and 27%, respectively), followed by the HMB only (10% and 14%, respectively), UF only (8% and 11%, respectively), and control (4% and 4%, respectively) cohorts.

### Treatment utilization and patterns

**FIG. 1.**
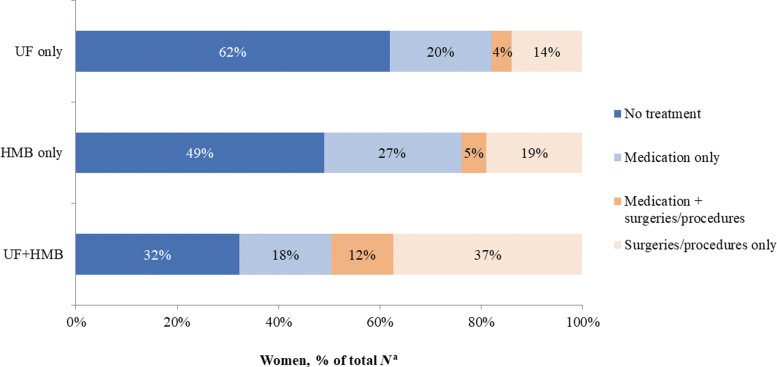
UF/HMB-related treatment during the post-index period in the matched cohorts. ^a^Proportions are based on *n* = 209,248 women in each matched cohort. HMB, heavy menstrual bleeding; UF, uterine fibroids.

During the pre-index year, the majority in the matched cohorts with UF only (78%), HMB only (79%), and UF+HMB (72%) had received no UF/HMB-related treatments. Among treated women, those diagnosed with UF+HMB used contraceptives and other medications more often (19% and 10%, respectively) than women with UF only (16% and 5%, respectively) and HMB only (13% and 7%, respectively). During the post-index year, UF/HMB-related treatments were recorded in 67%, 51%, and 38% of women in the UF+HMB, HMB only, and UF cohorts, respectively (Fig. 1). Nearly half (49%) of women with UF+HMB had undergone surgeries/procedures either with (12%) or without (37%) medications. By comparison, women with HMB only and UF only were managed with surgeries/procedures less frequently (24% and 18%, respectively) and received medications only more often (27% and 20%, respectively).

**FIG. 2.**
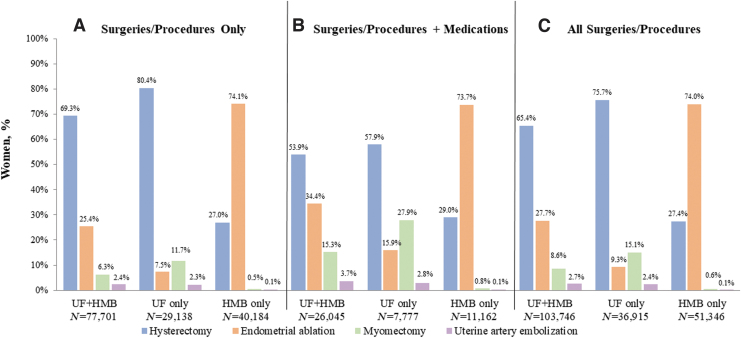
Post-index frequency of UF/HMB-related surgeries/procedures with and without medication use in the matched cohorts. Shown are percentages for cohorts of women that underwent **(A)** Surgeries/Procedures, **(B)** Surgeries/Procedures+Medications, or **(C)** All Surgeries/Procedures.

Figure 2 shows the proportions of women in each cohort who were treated with different types of surgeries and procedures only (Fig. 2A), surgeries, procedures, and medications (Fig. 2B), and irrespective of medication use in the overall population (Fig. 2C). Regardless of medication use, hysterectomy was the most frequently utilized procedure in the cohorts with UF only (76%; 27,923/36,915) and UF+HMB (65%; 67,902/103,746), whereas endometrial ablation was the most common procedure in the HMB only cohort (74%; 38,014/51,346) (Fig. 2C). Age differences among the surgery/procedure subgroups within the cohorts were not apparent with the exception of the subgroups with UF+HMB. Women with UF+HMB who underwent hysterectomies (*n* = 52,270) tended to be older than those who received myomectomies (*n* = 3,693); 81% in the former cohort were aged 39–51 years, whereas the majority (59%) in the latter were in their late 20s or 30s. Utilization of post-index surgeries/procedures among women with anemia appeared similar to the utilization in the overall study cohorts without anemia during baseline.

**FIG. 3.**
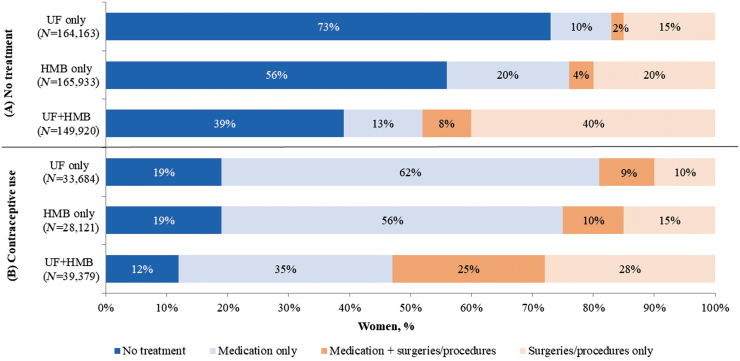
Post-index UF/HMB-related treatment in the matched cohort subgroups with **(A)** no treatment and **(B)** contraceptive use at baseline.

Examining treatment patterns among the respective subgroups with (1) no treatments and (2) contraceptive use before diagnosis (Fig. 3), treatment was initiated most often after the diagnosis of UF+HMB (61%; 90,985/149,920) and less often following the diagnosis of HMB only (44%; 73,289/165,933) and UF only (27%; 44,866/164,163). Surgeries and procedures were utilized most frequently in the previously untreated cohort with UF+HMB (48%) than in the HMB only (24%) and UF only (17%) cohorts. Following diagnosis, higher proportions of previously untreated women with HMB only were managed with medications only than those with UF only (20% vs. 10%, respectively). Among the subgroups with baseline contraceptive use, the UF+HMB subgroup featured the highest proportion (53%) treated with surgeries/procedures, followed by the HMB only (25%) and UF only (19%) subgroups, and was treated with only medications less often (35% vs. 56% and 62% in the HMB only and UF only subgroups, respectively). In the UF+HMB subgroup using contraceptives before diagnosis, 12% had no treatments during the post-index period compared with 19% each in the UF only and HMB only subgroups.

### Health care costs in the post-index year

**FIG. 4.**
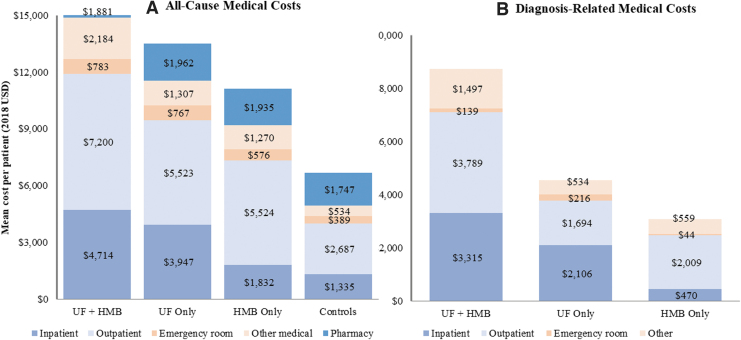
Mean all-cause **(A)** and diagnosis-related medical costs **(B)** during the post-index year. Mean costs are based on *n* = 209,248 in each matched cohort. All pairwise differences in total costs, inpatient, and outpatient costs were statistically significant (*p* < 0.05).

Approximately 5% of women in the matched cohorts had ≥1 inpatient admission during the pre-index period. During the post-index year, the UF+HMB cohort experienced the highest frequency of inpatient admissions (22%), followed by the cohorts with UF only (18%), HMB only (7%), and neither diagnosis (5%). The mean all-cause total cost for the UF+HMB cohort ($16,762 ± $25,398) exceeded that for the cohorts with UF only ($13,506 ± $28,242) and HMB only ($11,135 ± $24,946) by 24% and 50%, respectively, and was more than twice the mean cost for the control cohort ($6,691 ± $22,017) (all, *p* < 0.001) (Fig. 4 and Supplementary Appendix Tables SAT1 and SAT2). The mean diagnosis-related medical cost for UF+HMB ($8,741 ± $11,460) was 92% higher than that for UF only ($4,550 ± $10,509) and nearly triple the mean cost for HMB only ($3,081 ± $6,775) (all, *p* < 0.0001).

Over half (59%) of the medical costs incurred by women with UF+HMB were diagnosis-related compared with 39% and 33% of women with UF only and HMB only, respectively. Diagnosis-related inpatient and outpatient costs accounted for higher proportions of the respective all-cause component costs in the UF+HMB cohort (70% and 53%, respectively) than in the UF only (53% and 31%, respectively) and HMB only (26% and 36%, respectively) cohorts. Similarly, UF/HMB-related surgery/procedure costs represented higher percentages of the total all-cause and diagnosis-related medical costs in the UF+HMB cohort (47% and 80%, respectively) compared with the cohorts with UF only (24% and 61%, respectively) and HMB only (24% and 72%, respectively) (Supplementary Appendix Fig. SAF2). The mean surgery/procedure cost was also significantly higher for the UF+HMB cohort ($7,016 ± $10,631) compared with the cohorts with UF only ($2,763 ± $8,429) and HMB only ($2,217 ± $5,505) (all, *p* < 0.0001).

Pharmacy costs comprised a larger proportion of total health care costs in the control cohort (26%) than in the cohorts with HMB only (17%), UF only (15%), and UF+HMB (11%). ANOVA with *post hoc* Tukey honestly significant difference pairwise comparisons showed that women with neither diagnosis on average incurred significantly higher pharmacy costs compared with the other cohorts (all, *p* < 0.0001), whereas women diagnosed with UF only had significantly higher mean pharmacy costs than those with UF+HMB ($1,962 ± $9,722 vs. $1,881 ± $8,168; *p* = 0.0153).

## Discussion

Asymptomatic UF have been reported in 50%–75% of cases and may be detected incidentally during routine examinations or pelvic imaging.^15^ When clinical manifestations occur, HMB is the most common and often troubling symptom that can bear debilitating effects on health, daily activities, and quality of life.^4^ While women with UF who experience HMB may be expected to carry a higher disease burden than asymptomatic cases, data on the incremental cost impact are lacking. To the best of our knowledge, this is the first study to examine the direct health care costs and treatment patterns of women in the United States who were diagnosed with either UF or HMB or both conditions.

In the study population of nearly 3.4 million women of reproductive age, the cumulative incidence of UF diagnosed over the 11-year period was 15%. HMB was diagnosed in over half (54%) of these cases. Overall, the UF+HMB cohort represented an older population; nearly 80% of women were aged ≥39–51 years. In contrast, women aged ≥39–51 years account for 36% of women aged 18–51 years in the general U.S. population.^16^ Among the cohorts, anemia was most prevalent among the women with UF+HMB. These cases exhibited nearly twice the pre- and post-index rates of anemia compared with women who experienced HMB without a diagnosis of UF. Compared with those with UF only, women with UF+HMB incurred excess mean total costs of 24% ($16,762 vs. $13,506) during the post-index year. A prior claims analysis of the MarketScan database (January 2010‒December 2014) cited all-cause total surgical costs (including inpatient, outpatient, and medical surgery-related costs) averaging $15,813 ± $13,804 (2017 USD) for 95,433 commercially insured women with UF (mean age, 44.3 ± 6.4 years).^13^ Although the analysis did not differentiate between the costs for UF cases with and without a HMB diagnosis, the mean total cost appeared to approximate the estimated mean total health care costs for women with UF+HMB and UF only observed in the present study.

As previously reported,^11^ UF/HMB-related costs were primarily driven by the utilization of surgeries/procedures, particularly hysterectomy, and represented as much as 80% of the diagnosis-related medical costs for UF+HMB. About half of the women with UF+HMB treated with contraceptives before diagnosis underwent ≥1 surgery/procedure during the post-index year, suggesting heavier symptomatology or the need for a more effective management approach. Despite lower utilization of surgeries/procedures in the UF only (18%) and HMB only (24%) cohorts (which may reflect clinical recommendations for first-line pharmacotherapy^17,18^ or a high proportion of untreated asymptomatic UF cases), the associated costs accounted for the majority (61% and 72%) of diagnosis-related costs.

A majority of women with UF undergo ≥1 surgical or diagnostic procedure in the year following diagnosis,^19^ while the post-index year generally represents the peak period for pharmacological treatments for women managed nonsurgically.^5,20^ Examination of pre- and post-index treatment utilization revealed that the use of UF/HMB treatments increased following diagnosis, but only a modest proportion (38%) with UF only were treated. Some UF cases may have been asymptomatic and followed *via* an expectant management approach.^15^ The underlying reasons, however, could not be ascertained from the claims data. Other factors, such as patient preferences and behaviors in seeking medical care, fertility concerns, and variations in clinical practice, treatment options, and access to women's health care specialists, may have also influenced treatment decisions^21^ and merit future investigation.

Including the HMB only cohort allowed examination of cost differences between UF with HMB and HMB due to other causes. Overall, treatment for HMB only was less costly than UF regardless of a concomitant diagnosis of HMB. This may be attributable to the comparatively higher mean costs for inpatient admissions and surgeries/procedures in the UF cohorts. Compared with the control cohort, however, managing women with HMB only increased average total health care costs in the post-index year by 66% ($11,135 vs. $6,691, *p* < 0.0001). This finding appeared consistent with a prior study demonstrating a 68% increase in the mean costs for women with HMB versus controls ($6,439 ± $8,682 vs. $3,832 ± $8,308; *p* < 0.01).^18^

### Strengths and limitations

Several limitations are noted. First, the findings pertained to a commercially insured population in the United States and therefore may not be generalizable to patients who are uninsured or insured by other payers (such as Medicaid). Identification of the cohorts, costs, and resource use also relied on diagnostic codes rather than rigorous formal assessments and may be subject to errors. Additionally, there are no diagnostic codes currently dedicated to HMB associated with UF, and therefore, a recorded diagnosis of HMB did not necessarily indicate HMB caused by UF. Furthermore, while costs were compared between matched cohorts, the claims data were not sufficiently detailed to control for all factors potentially affecting treatments and costs. For example, several studies have reported that black women are significantly more likely than white women to be diagnosed with UF at a younger age and experience more severe symptoms.^9,11,22,23^ However, data on race were not available. Possible related factors such as smoking or drinking habits were likewise not available in the database. Finally, the standard deviations for some mean cost values appeared large, suggesting variance in the data. Additional analyses adjusting for outliers may yield more precise estimates.

Other factors may have contributed to the underestimation of costs, but nevertheless have implications for future research. The retrospective claims-based nature of this study precluded consideration of undiagnosed UF cases, which may be prevalent^23^ and incur costs for symptomatic treatment. Additionally, the study analyzed only pharmaceutical and surgical/procedural interventions, whereas previous literature has shown that lifestyle factors, such as physical activity, dietary changes, and weight management, can mitigate the risk of UF^24^ and may bear cost implications.^24^ While the follow-up period was limited to 1 year, some studies suggest that the likelihood of hysterectomy among women with UF may increase over time.^25^

Strengths of this study include that the IBM MarketScan Commercial Claims and Encounters Database was used as the source for the patient electronic health records, which is a nationwide database that allowed us to include records from individuals of various geographical locations in the United States and races and ethnicities. Another advantage of this database is that it includes claims for mail order prescriptions and specialty drugs, such as injectables.

## Conclusions

UF complicated by HMB were associated with significantly higher direct health care costs compared with UF or HMB alone. While surgical and procedural costs contribute substantially to diagnosis-related medical costs, effective pharmacological strategies can alleviate the clinical burden of symptomatic UF, particularly HMB, by reducing surgeries and procedures, thus ameliorating associated health care costs.

## Supplementary Material

Supplemental data

Supplemental data

Supplemental data

Supplemental data

## Data Sharing Statement

AbbVie is committed to responsible data sharing regarding the clinical trials we sponsor. This includes access to anonymized, individual, and trial-level data (analysis data sets), as well as other information (*e.g.*, protocols and Clinical Study Reports), as long as the trials are not part of an ongoing or planned regulatory submission. This includes requests for clinical trial data for unlicensed products and indications.

This clinical trial data can be requested by any qualified researchers who engage in rigorous, independent scientific research and will be provided following review and approval of a research proposal and Statistical Analysis Plan and execution of a Data Sharing Agreement. Data requests can be submitted at any time and the data will be accessible for 12 months, with possible extensions considered. For more information on the process, or to submit a request, visit the following link: https://www.abbvie.com/our-science/clinical-trials/clinical-trials-data-and-information-sharing/data-and-information-sharing-with-qualified-researchers.html

## Supplementary Material

Supplementary Appendix Figure SAF1

Supplementary Appendix Figure SAF2

Supplementary Appendix Table SAT1

Supplementary Appendix Table SAT2
