# Supplementary material for: Health Care Costs and Treatment Patterns Associated with Uterine Fibroids and Heavy Menstrual Bleeding: A Claims Analysis
Source: J Womens Health (Larchmt). 2022 Jun 14;31(6):856–63. doi: 10.1089/jwh.2020.8983 (PMC9245789; doi:10.1089/jwh.2020.8983)
Supplement: Supplemental data [file Suppl_Appendix_FigureSAF1.docx]

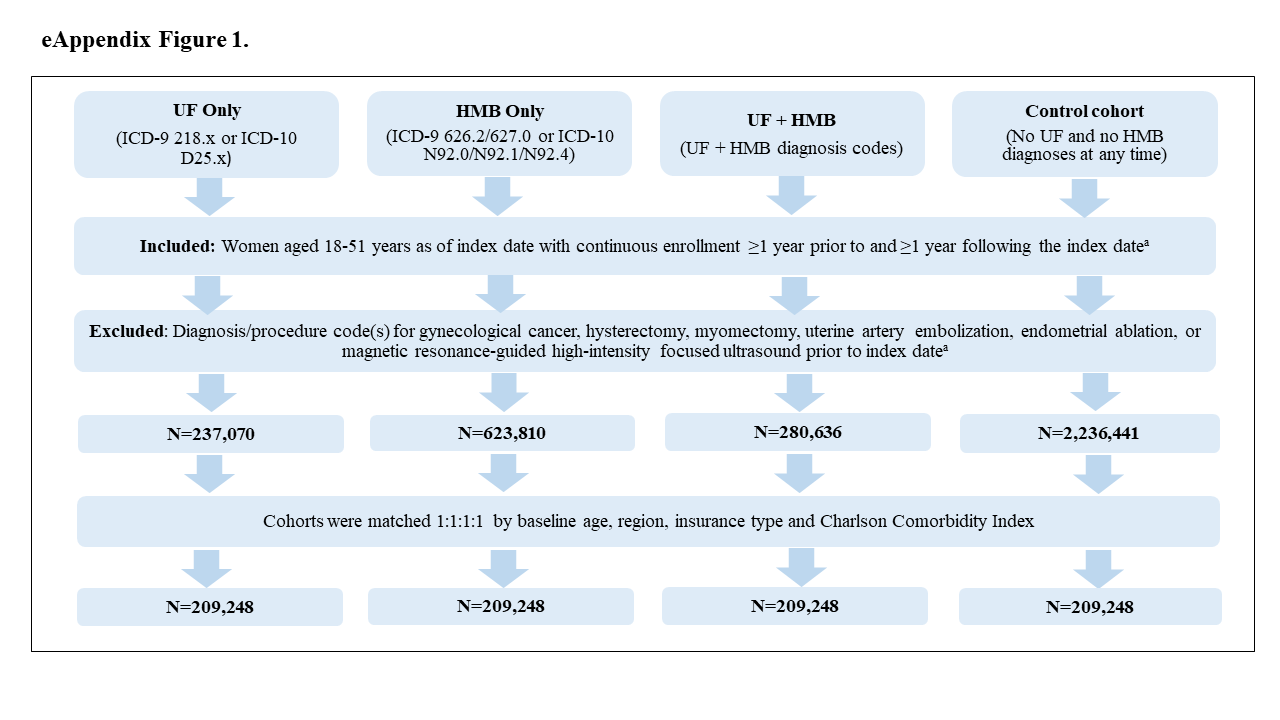


**Appendix Figure 1. Selection of Cohorts from the IBM MarketScan Database (10/1/2007‒9/30/2018).** ^a^Index date was defined as the date of first UF diagnosis for the UF cohort or HMB diagnosis for the HMB cohort, or the later date of first UF or first HMB diagnosis for the UF+HMB cohort. Index dates for the control cohort were randomly assigned from a uniform distribution in the study period. HMB indicates heavy menstrual bleeding; UF, uterine fibroid
