# Supplementary material for: Health Care Costs and Treatment Patterns Associated with Uterine Fibroids and Heavy Menstrual Bleeding: A Claims Analysis
Source: J Womens Health (Larchmt). 2022 Jun 14;31(6):856–63. doi: 10.1089/jwh.2020.8983 (PMC9245789; doi:10.1089/jwh.2020.8983)
Supplement: Supplemental data [file Suppl_Appendix_TableSAT1.docx]

**eAppendix Table 1. Mean All-cause Direct Healthcare Costs in the Matched Cohorts During the Post-index Year^a^**

|  | Mean ± standard deviation | | | |  |
| --- | --- | --- | --- | --- | --- |
| Costs (2018 USD) | UF+HMB | UF only | HMB only | Controls | p-value^b^ |
| Total medical | $14,881 ± $22,479 | $11,544 ± $24,406 | $9,201 ± $21,521 | $4,944 ± $18,971 | <.0001 |
| Emergency room | $783 ± $2,897 | $767 ± $2,867 | $576 ± $2,403 | $389 ± $2,014 | <.0001 |
| Inpatient | $4,714 ± $15,992 | $3,947 ± $17,213 | $1,832 ± $15,757 | $1,335 ± $13,719 | <.0001 |
| Outpatient | $7,200 ± $10,591 | $5,523 ± $11,258 | $5,524 ± $8,997 | $2,687 ± $7,789 | <.0001 |
| Other costs | $2,184 ± $8,217 | $1,307 ± $6,772 | $1,270 ± $6,437 | $534 ± $5,149 | <.0001 |
| Pharmacy | $1,881 ± $8,168 | $1,962 ± $9,722 | $1,935 ± $8,925 | $1,747 ± $7,981 | <.0001 |
| Total health care | $16,762 ± $25,398 | $13,506 ± $28, 242 | $11,135 ± $24,946 | $6,691 ± $2,2017 | <.0001 |

^a^Mean costs were based on N=209,248 women in each cohort

^b^Differences in costs were assessed using analysis of variance. *P*<0.0001 indicates a statistically significant difference

HMB indicates heavy menstrual bleeding; UF, uterine fibroid; USD, United States dollar
