# Supplementary material for: Health Care Costs and Treatment Patterns Associated with Uterine Fibroids and Heavy Menstrual Bleeding: A Claims Analysis
Source: J Womens Health (Larchmt). 2022 Jun 14;31(6):856–63. doi: 10.1089/jwh.2020.8983 (PMC9245789; doi:10.1089/jwh.2020.8983)
Supplement: Supplemental data [file Suppl_Appendix_TableSAT2.docx]

**eAppendix Table 2. UF/HMB Diagnosis-related Medical Costs in the Matched Cohorts During the 12-Month Post-Index Period^a^**

^a^Mean costs were based on N=209,248 women in each cohort

^b^Differences in costs were assessed using analysis of variance. *P*<0.05 indicates a statistically significant difference

HMB indicates heavy menstrual bleeding; UF, uterine fibroid; USD, United States dollar

|  | Mean ± standard deviation | | |  |
| --- | --- | --- | --- | --- |
| Costs (2018 USD) | UF+HMB | UF only | HMB only | p-value^b^ |
| Total medical | $8,741 ± $11,460 | $4,550 ± $10,509 | $3,081 ± $6,775 | <.0001 |
| Emergency room | $139 ± $1,157 | $216 ± $1,335 | $44 ± $655 | <.0001 |
| Inpatient | $3,315 ± $8,944 | $2,106 ± $9,120 | $470 ± $4,870 | <.0001 |
| Outpatient | $3,789 ± $7,030 | $1,694 ± $4,461 | $2,009 ± $4,072 | <.0001 |
| Other costs | $1,497 ± $5,468 | $534 ± $3,385 | $559 ± $2,659 | <.0001 |
