# Supplementary material for: Health Care Costs and Treatment Patterns Associated with Uterine Fibroids and Heavy Menstrual Bleeding: A Claims Analysis
Source: J Womens Health (Larchmt). 2022 Jun 14;31(6):856–63. doi: 10.1089/jwh.2020.8983 (PMC9245789; doi:10.1089/jwh.2020.8983)
Supplement: Supplemental data [file Suppl_Appendix_FigureSAF2.docx]

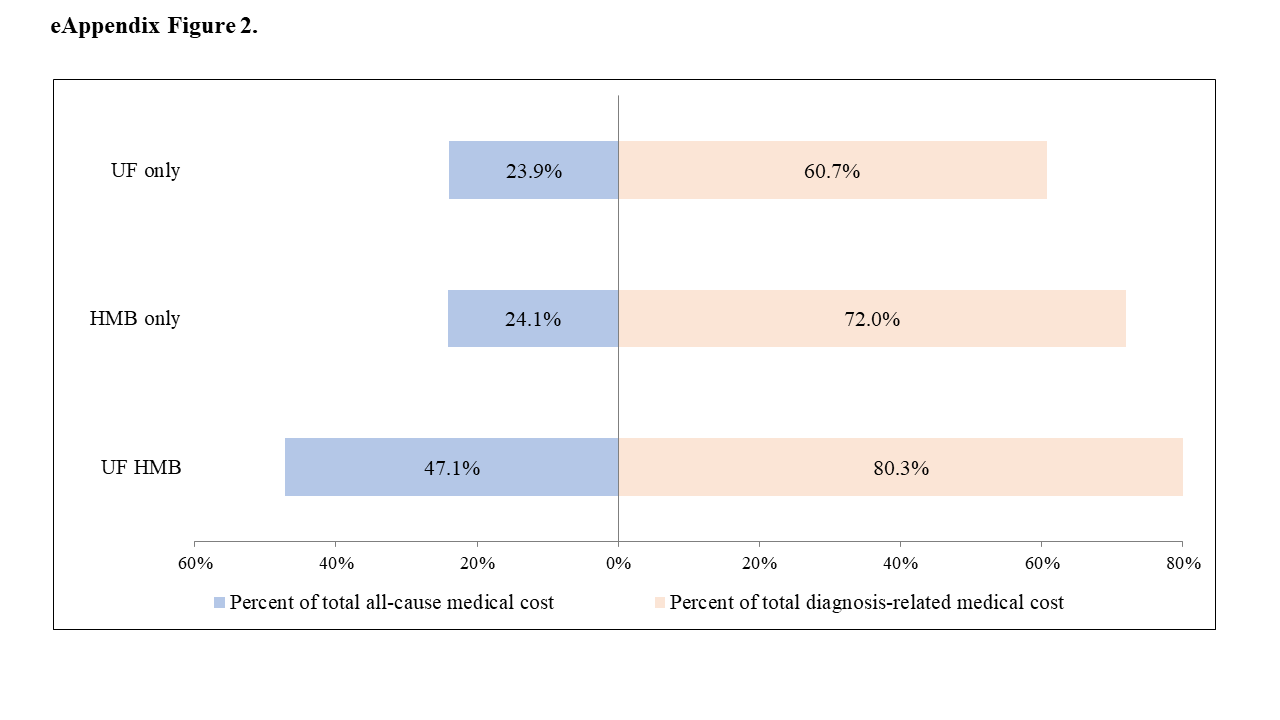


**Appendix Figure 2. Surgery/Procedure Costs as a Percentage of All-Cause and Diagnosis-related Medical Costs^a^.** ^a^Based on mean costs for N=209,248 women in each cohort

HMB indicates heavy menstrual bleeding; UF, uterine fibroid
